# Noncompliance With Treatment Regimens in a Patient With End-Stage Renal Disease: Complications, Socioeconomic Factors, and Management

**DOI:** 10.7759/cureus.84105

**Published:** 2025-05-14

**Authors:** George Mikhail, Sajia Mahmoodi, Sammy Matta, Roxana Lazarescu, Cho Zin

**Affiliations:** 1 Internal Medicine, St. George's University School of Medicine, New York, USA; 2 Internal Medicine, Wyckoff Heights Medical Center, New York, USA

**Keywords:** end-stage renal disease (esrd), focal segmental glomerulosclerosis (fsgs), medication noncompliance, public health and social work, sociodemographic disparity

## Abstract

In this case study, we examine a 27-year-old African American patient with a past medical history of hypertension, heart failure with mid-range ejection fraction (EF), and end-stage renal disease (ESRD) on hemodialysis, secondary to focal segmental glomerulosclerosis (FSGS). The patient has demonstrated noncompliance with his medications and hemodialysis, resulting in multiple emergency department visits and hospital readmissions. This report explores his medical challenges and evaluates strategies to improve care through a multidisciplinary, multisystem approach. Additionally, we examine the socioeconomic factors contributing to his noncompliance and discuss their broader implications for the healthcare system.

## Introduction

Chronic illnesses such as end-stage renal disease (ESRD) pose significant challenges, particularly when compounded by noncompliance with treatment regimens. In young adults, this issue is further complicated by the intersection of physical health, mental well-being, socioeconomic factors, and lifestyle pressures. Among psychosocial contributors, depression has been identified as the most prevalent factor associated with hemodialysis nonadherence [1]. This case study focuses on a 27-year-old African American male patient with ESRD, hypertension, heart failure with mid-range ejection fraction (EF), and a history of focal segmental glomerulosclerosis (FSGS). The incidence of FSGS has increased in recent years in the United States, with ESRD recognized as a major complication. FSGS is now the leading cause of nephrotic syndrome in the United States, with the highest prevalence among the Black population [2]. Although the underlying etiology remains unclear, genetic predisposition may play a role. Specifically, Black individuals are thought to have a lower nephron mass, which may contribute to an increased susceptibility to hypertension and subsequent glomerulosclerosis [3].

Despite receiving ongoing medical care, the patient's noncompliance with both hemodialysis sessions and prescribed medications has resulted in frequent hospitalizations, adverse health outcomes, and an overall decline in quality of life. Missing three or more hemodialysis sessions within a one-month period is associated with a 20% increased risk of mortality [4]. This case underscores the importance of a holistic, multisystem approach in managing chronic conditions such as ESRD. It highlights the need for integrated care strategies that address not only the medical concerns but also the social and psychological factors that impact patient adherence. Through this evaluation, we explore how effective patient management can be achieved by tailoring care plans to individual needs, fostering multispecialty collaboration, and addressing the broader socioeconomic barriers that influence treatment adherence.

## Case presentation

The patient is a 27-year-old young man with a medical history of ESRD on a thrice-weekly hemodialysis schedule. He was initially diagnosed in August 2023 following a renal biopsy, which revealed FSGS associated with an APOL1 gene variant. During his first documented encounter in May 2024, he presented with chest pain, shortness of breath (SOB), and severely elevated blood pressure (224/143 mmHg). He reported missing his scheduled hemodialysis session that day. The patient originally had an arteriovenous (AV) fistula, which became dysfunctional, necessitating the placement of a permacath for ongoing hemodialysis. He received three dialysis sessions during that hospitalization and was discharged with instructions to continue hemodialysis three times per week, along with an optimized antihypertensive regimen.

**Figure 1:**
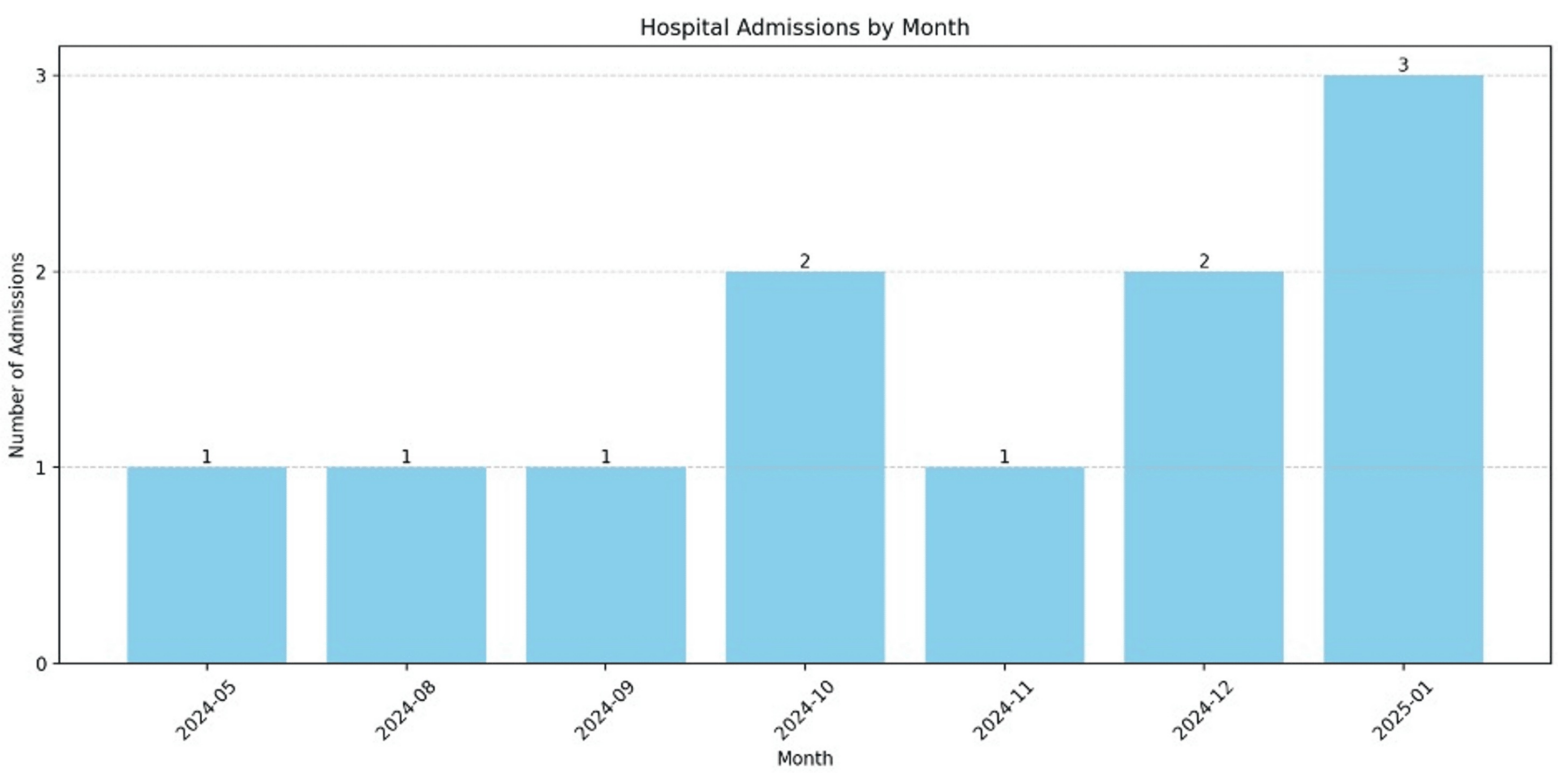
Hospital admissions by month

Over the past year, the patient has been admitted to the hospital on 10 separate occasions. The number of monthly admissions for 2024 is illustrated in Figure 1. Each admission was characterized by complaints of SOB and hypertensive episodes, consistently in the context of missed hemodialysis sessions. Complications identified during these visits included hypertensive emergencies, atelectasis, acute hypoxic respiratory failure secondary to fluid overload, and type 2 myocardial infarction (MI) due to demand ischemia. In each instance, the patient was stabilized, received hemodialysis, and was counseled on the importance of hemodialysis adherence and medication compliance. However, on multiple occasions, he left the hospital against medical advice.

**Figure 2:**
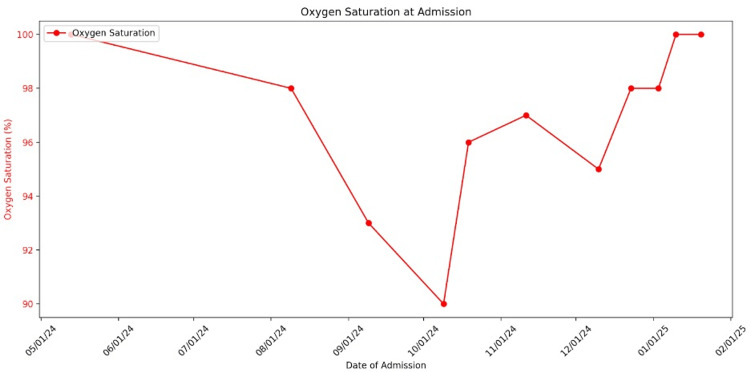
Oxygen saturation at admission

**Figure 3:**
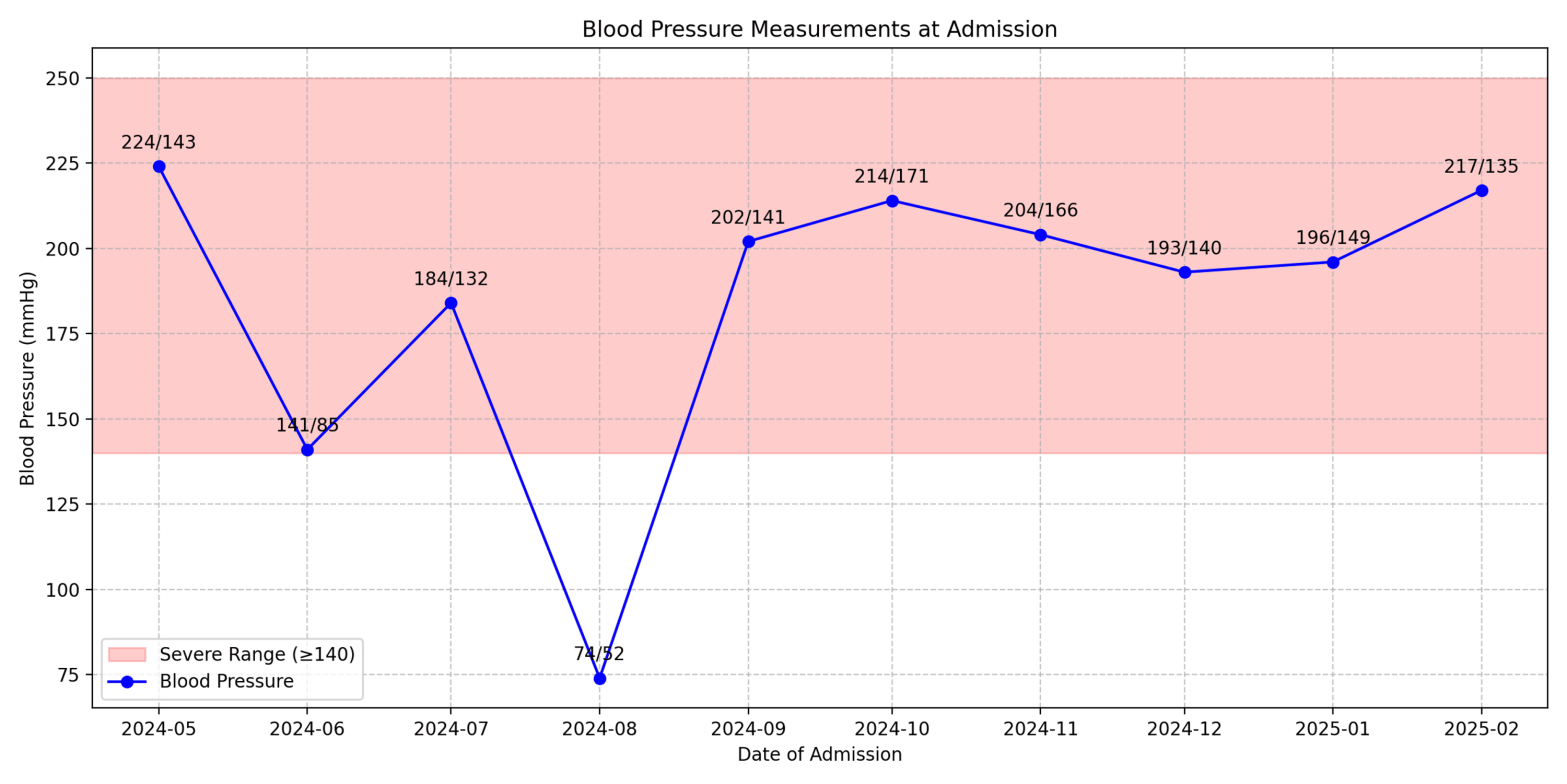
Blood pressure measurements at admission

During the patient's most recent admission in January 2025, he was brought in by EMS on a stretcher, receiving 2 L of oxygen via nasal cannula for SOB, with an oxygen saturation of 95%. Figure 2 illustrates the fluctuations in his oxygen saturation at the time of admission. He had again missed a scheduled hemodialysis session. On examination, bilateral rales were heard on auscultation, and his blood pressure was elevated at 196/149 mmHg. Figure 3 summarizes his elevated blood pressure readings on admission. The patient also reported abdominal pain, nausea, and vomiting. He was admitted with a diagnosis of acute hypoxic respiratory failure secondary to fluid overload due to missed hemodialysis. However, before further workup or initiation of treatment, the patient elected to leave the hospital against medical advice.

Although a formal psychological evaluation was not conducted, we acknowledge the critical importance of the patient’s mental health and potential extenuating life circumstances in influencing outpatient care and overall outcomes. Moving forward, we plan to incorporate a comprehensive psychosocial assessment as part of a multidisciplinary team approach to care planning and management.

Treatment and management

On his initial admission in 2024, the patient had a permacath placed and was prescribed hemodialysis three times a week. During his second admission in August 2024, he left the hospital against medical advice. On his third admission, he was discharged with an optimized antihypertensive medication regimen and follow-up appointments with cardiology, nephrology, and his primary care provider (PCP). During his fourth admission in October 2024, the patient presented with a three-day history of chest pain and SOB. He admitted to missing multiple hemodialysis sessions. Laboratory findings revealed metabolic acidosis, hyperkalemia, acute on chronic systolic heart failure, and hypoxia due to fluid overload. He was borderline hypotensive and desaturating, nearly progressing to cardiogenic shock. He was treated with BiPAP ventilation, IV labetalol, and emergent hemodialysis. At discharge, he was advised to follow up with appropriate specialty clinics, counseled on medication adherence, and prescribed aspirin 81 mg and atorvastatin 40 mg. On his seventh admission, the patient was prescribed carvedilol, which he failed to obtain. He was again counseled on the importance of medication and hemodialysis compliance. On the eighth admission, he was again advised on medication compliance and counseled to avoid smoking, alcohol, and recreational drug use. On the ninth admission, he again left the hospital against medical advice, as well as on the first admission of 2025. At his most recent discharge, the patient was advised to follow up with his outpatient PCP, cardiologist, and nephrologist. However, outpatient records show that his only follow-up was at a vascular clinic for AV fistula evaluation. His repeated failure to attend outpatient visits continues to compromise the effectiveness of his treatment and long-term care.

## Discussion

Noncompliance remains a widespread issue across clinical medicine, and in the context of hemodialysis, it significantly contributes to increased morbidity and mortality. This is much more evident in young adults, with studies showing that 88% of patients aged 19-34 are noncompliant, compared to 41% among those aged 35-79 [5]. In addition, 50% of all hemodialysis patients are nonadherent to various aspects of their treatment regimen [6]. Managing a chronic illness at a young age presents unique challenges. Young adults undergoing hemodialysis for chronic kidney disease often report lower quality-of-life scores, particularly in areas such as cognitive function, emotional well-being, and pain perception [7]. In addition to this age group's developmental and societal pressures, they must contend with the demands of a complex medical regimen.

In the case of ESRD, hemodialysis sessions are frequently missed, leading to hospitalizations. Hemodialysis typically requires three sessions per week, which can be difficult to maintain, especially for individuals balancing work, education, or family responsibilities. Also, for patients of lower socioeconomic statuses (SES), there might not always be an available, affordable method of transportation to take them to these sessions frequently. All these issues can cause disruptions and complicate a patient's daily life. This will not only lead to missed sessions but can also negatively impact their mental health.

Young adults are more likely to be uninsured and come from lower SES backgrounds, making them more vulnerable to poor health outcomes [8]. This vulnerability can create a cycle in which individuals with lower SES face greater limitations. Their ability to maintain employment is often hindered by the difficulty of balancing treatment demands with career responsibilities, further compounding their challenges. Studies have shown that lower SES is associated with worse outcomes in hemodialysis treatments. When data is stratified by race, studies indicate higher mortality rates among young African American patients [8]. A strong desire for normalcy is common among young adults, which can lead to denial or disregard of their chronic illness. In addition to denial and limited health education, mental health must also be considered. Notably, depression affects approximately 29% of patients undergoing hemodialysis [9].

In this case, the 27-year-old male patient with ESRD experienced multiple complications and required frequent hospitalizations due to treatment noncompliance, an issue commonly seen in younger patients. Serious chronic illnesses like ESRD present significant challenges, especially for young adults. The patient has a high school education, is currently unemployed, and lives with his family. Limited educational attainment may lead to uninformed decisions; thus, it is important not to make assumptions and to communicate in clear, accessible language. Employment and career aspirations often instill a sense of purpose, contributing to psychological well-being. Unfortunately, this sense of direction is lacking in our patient's case. Securing part-time employment that accommodates the demanding schedule of dialysis is challenging, and the physical burden of illness further complicates work opportunities. Although anti-discrimination laws exist, implicit biases in hiring practices may still pose barriers. These factors collectively contribute to poor disease management, often resulting in emotional and uninformed decisions. Patients may even forgo treatments known to improve outcomes, such as skipping dialysis sessions and missing outpatient appointments.

The LACE index is a clinical tool used to predict the risk of 30-day readmission. It takes into account the length of stay, acuity of admission, comorbidities, and recent emergency department visits. With a LACE score of 16, our patient falls into the high-risk category for readmission within 30 days of discharge [10]. To better support patients facing such challenges, the healthcare system should consider adapting its care guidelines to promote treatment adherence and improve overall outcomes. As demonstrated by this case, traditional approaches, such as prescribing medications and recommending follow-up visits, are not always effective. This is particularly true in lower socioeconomic communities, where health literacy and access to care may be limited.

This case highlights the need for updated strategies and guidelines to address noncompliance and the management of chronic diseases. A multidisciplinary team, which includes physicians, nurse managers, social workers, and case managers, is essential to achieving this goal. Ideally, such a team would meet daily to identify high-risk patients using tools like the LACE score. This risk stratification could then inform the development of individualized care plans tailored to better meet patients' needs and promote adherence to treatment regimens. 

A key advantage of a multidisciplinary team is its ability to address patients' needs after hospital discharge. Social workers, in particular, can connect patients with essential community resources, such as home health services, shelters, and transportation assistance. Another important recommendation is to schedule follow-up appointments within 7-10 days post-discharge. This continuity of care helps prevent complications and reduces the risk of readmissions by keeping patients engaged in their recovery process. Early follow-up visits enable healthcare providers to monitor progress, address concerns promptly, and reinforce care plans, ultimately optimizing patient outcomes.

For high-risk patients, such as the individual in this case, a strong social support system is a critical factor in promoting treatment compliance. An integral component of their care plan should involve actively engaging family members through education and ongoing support. Another promising initiative is the integration of telehealth and remote monitoring technologies. These tools enable regular check-ins and allow the healthcare team to track vital signs in real time, ensuring timely interventions and reducing the likelihood of hospital readmissions. This approach not only addresses medical needs but also helps overcome social barriers that may impede recovery. By fostering treatment adherence, minimizing complications, and decreasing hospital admissions, this model ultimately contributes to a significant improvement in patients’ overall quality of life.

Currently, a multidisciplinary care team approach is in place at Wyckoff Heights Medical Center. Each hospital unit is supported by a team comprising physicians, nurse managers, social workers, and case managers, who meet twice daily to discuss upcoming patient discharges and develop individualized post-discharge care plans. Although the available data is not yet sufficient for broad generalizations, anecdotal evidence suggests that this initiative has positively impacted patient compliance with outpatient care. By leveraging a coordinated team of healthcare professionals, the system is better equipped to address the complex needs of patients, especially those managing chronic conditions such as ESRD. This collaborative model fosters personalized care, facilitates smoother transitions from hospital to home, and ultimately promotes improved health outcomes and adherence to treatment regimens.

We acknowledge that alternative treatment options to conventional hemodialysis exist, some of which reduce the need for frequent clinic visits. However, our aim is not to provide a comprehensive review of these alternatives, but rather to underscore the importance of a holistic, individualized approach to outpatient post-discharge care. This case highlights the critical role such tailored care plays in supporting patient adherence and improving overall outcomes.

## Conclusions

One known complication of FSGS is the progression to ESRD, which often requires hemodialysis. Most young adults with chronic illnesses struggle to manage their treatments, often due to a range of social factors. Noncompliance frequently arises from limited health education or lower SES. The traditional approach of patient care (prescribing medications and scheduling follow-up appointments) is unsuitable for all situations. When a particular approach proves impractical for a patient, persisting with it is unlikely to yield improved outcomes. Instead, care strategies should be adapted using a multidisciplinary team that can tailor interventions to the individual's needs. In such cases, early involvement of social work and psychological services can be particularly valuable, as they address underlying barriers to adherence. Incorporating these services from the outset of hospitalization may help mitigate challenges patients face later in their care journey.

## References

[1] Hussain N, Hudson Saba Sile JQ, Sile S, Cowan P, Khan N, Acchiardo S (2001). Psychosocial factors and noncompliance in hemodialysis (HD) patients (PTS). Am J Kidney Dis.

[2] Kitiyakara C, Eggers P, Kopp JB (2004). Twenty-one-year trend in ESRD due to focal segmental glomerulosclerosis in the United States. Am J Kidney Dis.

[3] Kitiyakara C, Kopp JB, Eggers P (2003). Trends in the epidemiology of focal segmental glomerulosclerosis. Semin Nephrol.

[4] Leggat JE Jr, Orzol SM, Hulbert-Shearon TE, Golper TA, Jones CA, Held PJ, Port FK (1998). Noncompliance in hemodialysis: predictors and survival analysis. Am J Kidney Dis.

[5] Gonsalves-Ebrahim L, Sterin G, Gulledge AD, Gipson WT, Rodgers DA (1987). Non-compliance in younger adults on hemodialysis: younger adults are likely to need more assistance to ensure compliance. Psychosomatics.

[6] Ibrahim S, Hossam M, Belal D (2015). Study of non-compliance among chronic hemodialysis patients and its impact on patients' outcomes. Saudi J Kidney Dis Transpl.

[7] Tong A, Wong G, McTaggart S (2013). Quality of life of young adults and adolescents with chronic kidney disease. J Pediatr.

[8] Johns TS, Estrella MM, Crews DC (2014). Neighborhood socioeconomic status, race, and mortality in young adult dialysis patients. J Am Soc Nephrol.

[9] Adejumo OA, Edeki IR, Oyedepo DS (2024). Global prevalence of depression in chronic kidney disease: a systematic review and meta-analysis. J Nephrol.

[10] Rajaguru V, Han W, Kim TH, Shin J, Lee SG (2022). LACE index to predict the high risk of 30-day readmission: a systematic review and meta-analysis. J Pers Med.

